# Macrophage-based pathogenesis and theranostics of vulnerable plaques

**DOI:** 10.7150/thno.105256

**Published:** 2025-01-02

**Authors:** Fei Fang, Erxiang Wang, Mengjia Fang, Hongyan Yue, Hanqiao Yang, Xiaoheng Liu

**Affiliations:** 1 Institute of Biomedical Engineering, West China School of Basic Medical Sciences & Forensic Medicine, Sichuan University, Chengdu 610041, China.; 2 College of Veterinary Medicine, Northwest A&F University, Yangling 712100, Shaanxi, China.

**Keywords:** vulnerable plaque, macrophage, theranostics, nanoparticles

## Abstract

Vulnerable plaques, which are high-risk features of atherosclerosis, constitute critical elements in the disease's progression due to their formation and rupture. Macrophages and macrophage-derived foam cells are pivotal in inducing vulnerability within atherosclerotic plaques. Thus, understanding macrophage contributions to vulnerable plaques is essential for advancing the comprehension of atherosclerosis and devising novel therapeutic and diagnostic strategies. This review provides an overview of the pathological characteristics of vulnerable plaques, emphasizes macrophages' critical role, and discusses advanced strategies for their diagnosis and treatment. It aims to present a comprehensive macrophage-centered perspective for addressing vulnerable plaques in atherosclerosis.

## 1. Introduction

Atherosclerosis is a chronic inflammatory disease of blood vessels, representing a major cause of cardiovascular disease (CVD) on a global scale. This condition is marked by the development of atheroma and fibrous plaques within the intimal layer of blood vessels 1. Although age-related mortality from atherosclerosis has declined in recent years, it remains a leading global cause of death 2. Atherosclerosis progresses via complex mechanisms involving lipid accumulation, fibrous tissue proliferation, and plaque formation. These plaques result in thickening of the arterial lining, lumen narrowing, and arterial wall hardening, culminating in restricted blood flow to vital organs. During disease progression, plaques may detach from the arterial wall, forming thrombi that can migrate to other organs and cause severe complications, including strokes and myocardial infarction 3. Atherosclerotic plaques prone to thrombosis or rupture, referred to as unstable or vulnerable plaques, are characterized by a lipid-rich necrotic core and a thin fibrous cap infiltrated by macrophages 4. Vulnerable plaques substantially elevate the risk of thrombus formation, leading to strokes and heart attacks 5, 6. Vulnerable plaques substantially elevate the risk of thrombus formation, leading to strokes and heart attacks 7.

The progression of atherosclerosis involves a complex interplay of cellular mechanisms, including inflammation of endothelial cells (ECs), phenotypic changes in vascular smooth muscle cells (VSMCs), macrophage inflammatory responses, lipid transport dysfunction, and immune cell activation 8. Macrophages are pivotal in the development and progression of atherosclerosis, particularly in the growth and rupture of plaques 9. Within atherosclerotic plaques, macrophages exhibit diverse phenotypes, including classical M1 and M2 types as well as other subtypes such as Mox and M4 10, 11. From a traditional perspective, inflammatory M1 macrophages release pro-inflammatory factors that promote extracellular matrix (ECM) remodeling and VSMC proliferation, leading to lipid-laden plaque formation beneath the endothelium 12. Foam cells, which are lipid- and cholesterol-rich macrophages with impaired lipid metabolism, contribute to plaque rupture 13. Under normal conditions, macrophages phagocytose oxidized low-density lipoprotein (ox-LDL) through scavenger receptors (SR), such as LOX-1, CD36, and SR-A, which degrade cholesteryl esters into free cholesterol and free fatty acids within lysosomes. Free cholesterol is subsequently exported via ATP-binding cassette (ABC) transporters ABCA1, ABCG1, and SR-BI 14. However, this critical balance of cholesterol transport is disrupted in atherosclerotic conditions. Upregulated SR increases ox-LDL uptake, while suppressed ABCA1 and ABCG1 expression reduces cholesterol efflux, resulting in foam cell formation 14. Foam cells secrete matrix-degrading enzymes, facilitating fibrous cap detachment from the endothelium and thrombus formation 15. The presence of foam cells in plaques is strongly linked to increased plaque rupture risk 16. Given macrophages' pivotal role in vulnerable plaques, comprehensively understanding their pathological mechanisms is essential for effective atherosclerosis management.

This review aims to summarize the main pathological processes, *in vivo* mouse models, and macrophage-based insights into atherosclerotic vulnerable plaques. Additionally, it examines current strategies targeting macrophages or foam cells for the diagnosis and treatment of vulnerable plaques. This work provides a macrophage-centered perspective on diagnosing and treating vulnerable plaques, contributing to a deeper understanding of their pathophysiology and management.

## 2. Pathologic features and animal model

### 2.1. Pathologic features

**Figure 1:**
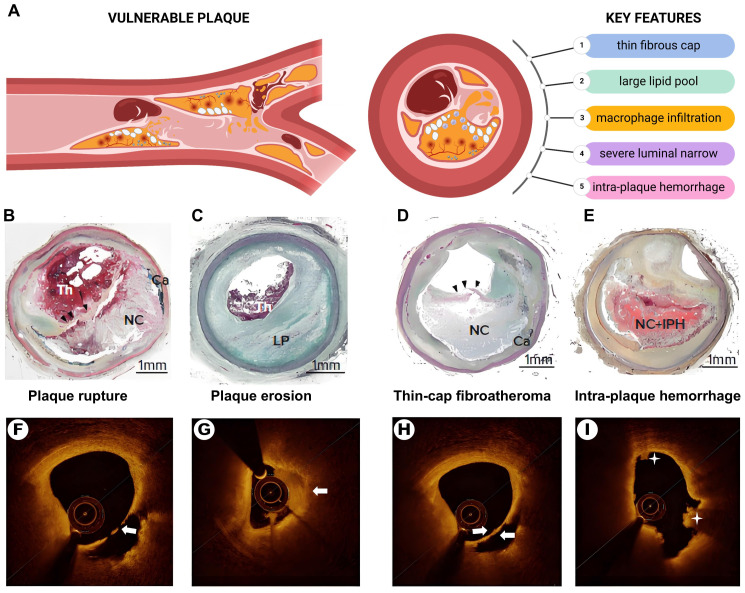
** The pathologic features of vulnerable plaques.** (A) The Schematic diagram of vulnerable plaque and its main pathological features. (B) plaque rupture. (C) plaque erosion with a large lipid pool. (C) Thin-cap fibroatheroma (black arrowhead). (E) intra-plaque hemorrhage. (F) Optical coherence tomography (OCT)-plaque rupture. (G) OCT-plaque erosion (white arrow shows thrombus). (H) Thin-cap fibroatheroma (white arrows show thin-cap fibroatheroma). (I) Intra-plaque hemorrhage (asterisk). Th = *thrombus*; NC = *necrotic core*; Ca = *calcification*; LP = *lipid pool*; IPH = *intra-plaque hemorrhage. Source: Panels B-E: Sakamoto et al., 2022 21. Adapted with permission from US Cardiology. Panels F-I: Xiaoxiao et al., 2021 22. Adapted with permission from Springer Nature. Panel A created with BioRender.com*.

The concept of vulnerable plaques, characterized by their propensity for rupture and thrombosis, was introduced by Muller *et al.* in the 1980s 17. Vulnerable plaques exhibit distinct anatomical, biological, and pathophysiological features. Autopsy studies have revealed key histological features linked to an increased risk of plaque rupture and acute coronary events (Figure 1). Key features include: 1) a thin fibrous cap (<65 μm); 2) a large lipid core (>40% of the plaque area); 3) pronounced macrophage infiltration; 4) endothelial cell ablation with platelet aggregation; and 5) severe luminal narrowing (>90%) 18-20. Secondary pathological features include neovascularization, vascular remodeling, superficial nodal calcification, endothelial dysfunction, enlarged necrotic cores, inflammation, and intraplaque hemorrhage.

### 2.2. Animal models

**Figure 2:**
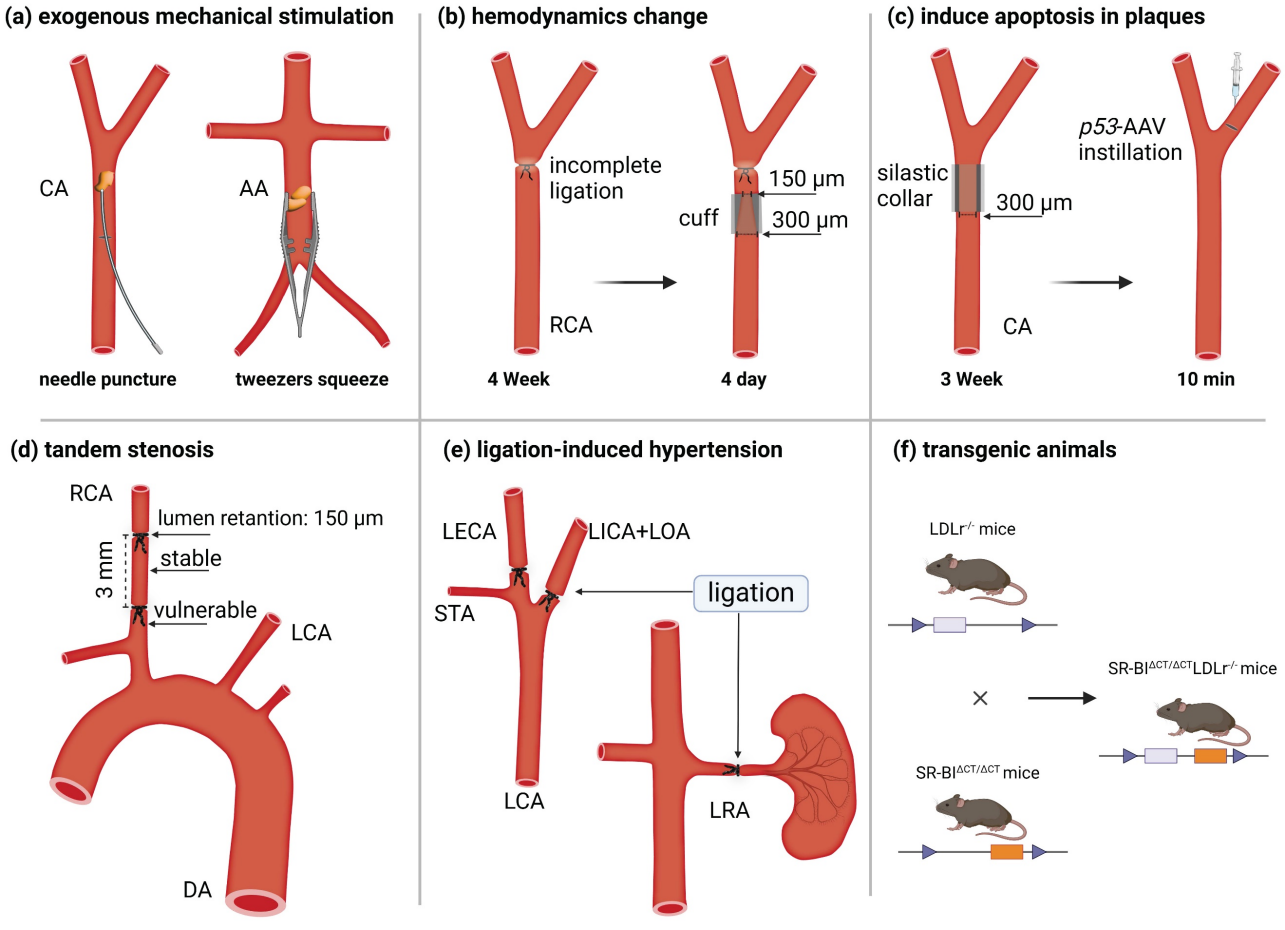
** Schematic diagram of the scheme for constructing a vulnerable plaque model in mice.** (a) Exogenous mechanical force destroys the plaque structure and causes plaque rupture, such as puncturing the plaque with a needle or squeezing the plaque with tweezers. (b) Plaques were formed after ligation, and plaque rupture was induced by changing hemodynamics by implanting a conical cuff. (c) After the implantation of a silicone collar induced plaque formation, plaque rupture was induced by the instillation of a virus that promotes cell apoptosis. (d) Tandem stenosis induces stable plaque and vulnerable plaque models by changing hemodynamic parameters. (e) Unilateral carotid and renal artery ligation induce hypertension and plaque rupture. (f) Transgenic animal hybrids.CA = *carotid artery*; AA = *abdominal aorta*; LCA = *left carotid artery*; RCA = *right carotid artery,* DA = *descending aorta,* LICA = *left internal carotid artery, LOA* = *left occipital artery, LECA* =* left external carotid artery, STA* =* superior thyrotropin artery, LRA* =* left renal artery. Figure created with BioRender.com.*

Transgenic ApoE^-/-^ and LDLr^-/-^ mice are widely used as models for experimental atherosclerosis research due to their accessibility and reproducibility. They are widely used as models for experimental atherosclerosis research due to their accessibility and reproducibility. Three primary methods are used in mouse models to induce plaque rupture. The first approach involves surgical disruption of the fibrous cap. For instance, Robert *et al.* used round-tipped tweezers to gently compress the abdominal aorta plaque of ApoE^-/-^ mice 23. Similarly, Jacob *et al.* punctured the carotid plaque with a 9-0 needle, causing rupture 24 (Figure 2A). The second method exploits hemodynamics alterations to destabilize plaque. For example, researchers performed incomplete ligation near the right carotid artery bifurcation in 12- to 14-week-old male mice to establish stable lesions. They then positioned conical polyethylene cuffs (300 and 150 μm diameters) over the ligature for 4 days, promoting collagen degradation and plaque rupture 25, 26 (Figure 2B). Jan *et al.* accelerated atherosclerosis in ApoE^-/-^ and LDLr^-/-^ mice by inserting a 0.3 mm inner diameter annular silicone ring into the common carotid artery for 3 weeks. After ring removal, adenovirus containing tumor suppressor protein p53 was injected into the distal artery and left to soak for 10 minutes. This approach targeted the delivery of p53 to VSMCs in the fibrous cap, inducing apoptosis within one day and triggering plaque rupture 27 (Figure 2C). Peter *et al.* created a vulnerable plaque model by double ligating the carotid artery at 1 mm and 4 mm from the upper bifurcation, following the insertion of a 150-µm needle beneath the vessel (Figure 2D) 28. This method produces two types of plaque in the right carotid artery: a stable plaque between the ligature points and a vulnerable plaque near the second ligature point. Finally, Jin *et al.* developed a spontaneous plaque rupture model with luminal thrombosis by partially ligating the left carotid artery and renal arteries in ApoE^-/-^ mice. Simultaneously, they activated the renin-angiotensin system to induce local stress changes (Figure 2E) 29.

The third method involves engineering transgenic mouse models specifically designed to develop vulnerable plaques. For example, Tian *et al.* generated vulnerable plaque model mice by crossbreeding Fbn1C1^039G+/-^ mice with LDLr^-/-^ mice 30 (Figure 2F). Wu *et al.* induced vulnerable plaques in NR1D1^-/-^ mice on a C57BL/6 background by partially ligating the left renal and carotid arteries 31. Recently, Shamsuzzaman *et al.* created an advanced atherosclerotic vulnerable plaque model by knocking out SR-BI in LDLr^-/-^ mice and administering a high-fat diet for 26 weeks 32.

## 3. The role of macrophages in vulnerable plaques

**Figure 3:**
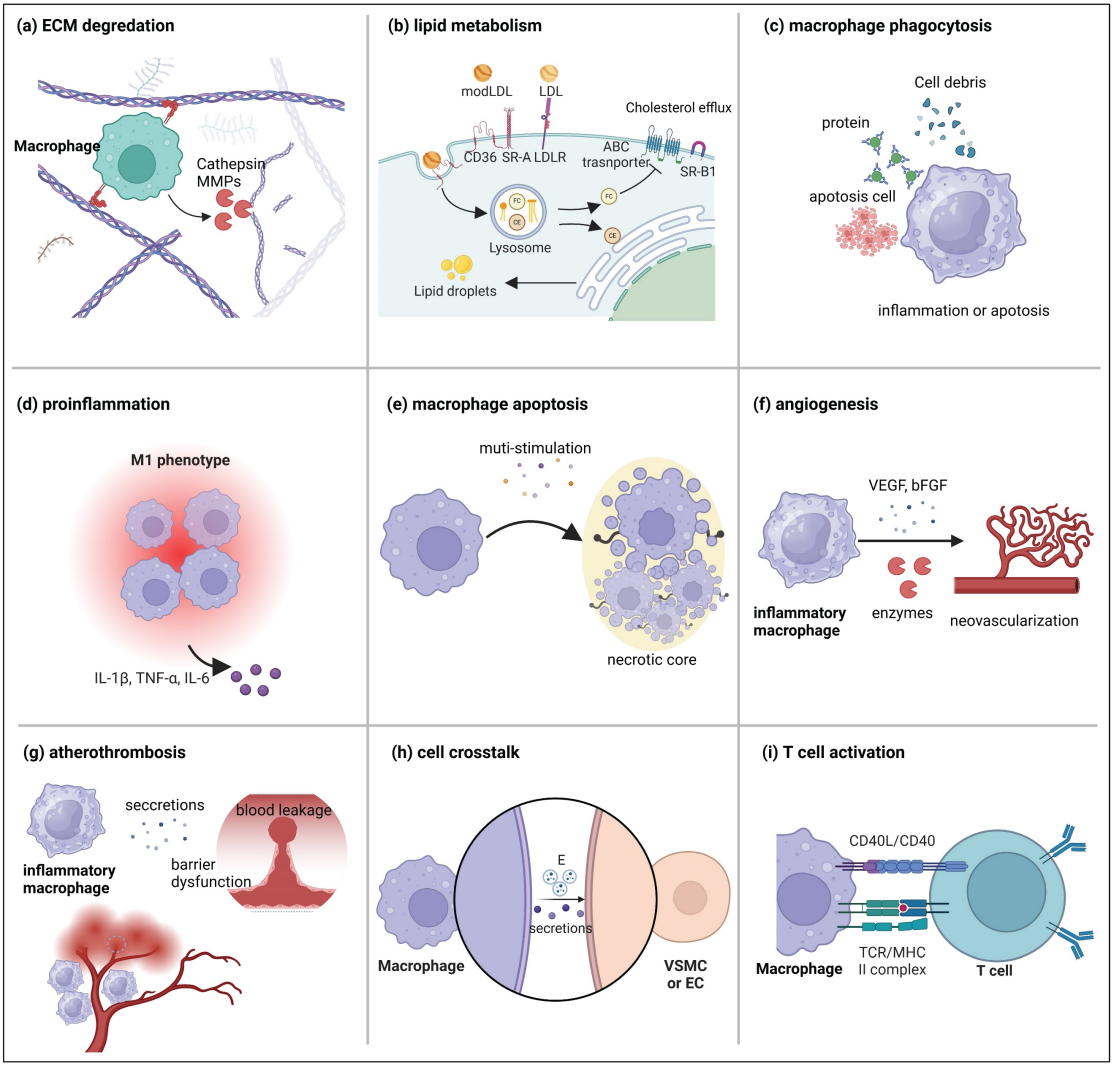
** The leading role of the macrophage in vulnerable plaque.** (a) Macrophages release enzymes such as MMPs and cathepsin to degrade the ECM. (b) Macrophage trafficking and metabolism of ox-LDL in plaques. (C) Macrophages phagocytize cell debris, proteins, and apoptotic cell components. (d) Macrophages secrete inflammatory factors to promote plaque inflammation. (e) Macrophage apoptosis in plaques. (f) Macrophages secrete VEGC and bFGF to promote angiogenesis. (g) Macrophages promote intraplaque hemorrhage. (h) Macrophages promote the phenotypic transformation of VSMCs and endothelial dysfunction. (i) Macrophages activate T cells through antigen presentation. *Figure created with BioRender.com.*

Although the role of macrophages in atherosclerotic plaque formation has been extensively reviewed elsewhere 33, this section emphasizes their contribution to plaque destabilization. This discussion highlights key macrophage functions in vulnerable plaques, including ECM remodeling, foam cell formation, inflammation, necrotic core formation, intra-plaque angiogenesis, intra-plaque hemorrhage, and interactions with other cell types (Figure 3).

### 3.1. ECM remodeling

The ECM is a complex, vital structure surrounding cells, crucial for maintaining tissue integrity and offering cellular support and protection 34. It comprises collage, non-collagen proteins, elastin, proteoglycans, and glycosaminoglycans. Collagen, a key component, provides tissue strength and flexibility, which is critical in safeguarding tissue integrity from damage 35. Studies indicate that ECM composition in vulnerable plaques undergoes significant changes, notably a marked reduction in collagen fiber content, a hallmark characteristic 36. The reduction in collagen is primarily attributed to the macrophages, the predominant cell types in plaques responsible for ECM degradation. Inflammatory macrophages and macrophage-derived foam cells within plaques secrete enzymes that degrade the ECM, contributing to plaque rupture 37 (Figure 3A).

Matrix metalloproteinases (MMPs) are key enzymes that regulate atherosclerotic plaque stability. Studies dating back to 1994 have shown increased expression of MMPs and heightened matrix degradation activity in vulnerable regions of human atherosclerotic plaques 38. Macrophage-associated MMPs that regulate plaque stability include collagenases (MMP-1, MMP-8, MMP-13), gelatinase (MMP-9), stromelysin (MMP-3, MMP-10), and other MMPs (MMP-7, MMP-12). Studies confirm that T cell-mediated adaptive immunity activates macrophages via CD40 signals, which upregulate the expression of collagenases MMP-1, MMP-8, and MMP-13 39. mRNA analysis of plaques from carotid endarterectomy patients showed that MMP-1 expression in plaques with thin fibrous caps was 7.8 times higher than in those with thick fibrous caps 40. Recent studies also show that MMP-1a co-expresses with PAR1 (protease-activated receptor-1) on monocytes, promoting monocyte/macrophage infiltration 41.

The primary gelatinase secreted by macrophages and foam cells in plaques is MMP-9, also called gelatinase B. Studies have shown that MMP-9 co-localizes with macrophages in unstable carotid plaques 42. Moreover, overexpression of the rabbit MMP-9 gene under the control of the scavenger receptor A (SR-A) enhancer/promoter resulted in more significant plaque burden and increased monocyte/macrophage infiltration 43; in contrast, ApoE/MMP-9 double knockout mice exhibited reduced aortic plaque burden, macrophage infiltration, and collagen content 44. MMP-3 (stromelysin 1) and MMP-10 (stromelysin 2) are among the earliest stromelysins identified in atherosclerotic plaques, with their expression localized to macrophages. Studies have shown that induced hypoxia upregulates MMP-3 and MMP-10 expression in macrophages via HIF-1α signaling, affecting the stability of mouse plaques 45.

In addition to MMPs, cysteine protease cathepsins (CTSs) play a crucial role in the ECM remodeling of atherosclerotic plaques. The cysteine protease family comprises 11 human members, including cathepsins B, C, H, F, K, L, O, S, V, W, and X/Z. Notably, cathepsins B 46, C 47, K 48, L 49, S 50, and V 51 have all been identified in atherosclerotic plaques. Cathepsin K (CTSK) is a potent type I collagen-degrading enzyme, and its expression is significantly upregulated in advanced plaques 48, 52, 53. A recent study elucidated that macrophages, particularly foam cells, are the primary producers of CTSK 54. Mechanistic studies have revealed that cholesterol loading in macrophages activates Toll-like receptor (TLR) signaling, leading to sustained phosphorylation of p38 mitogen-activated protein kinase and induction of CTSK expression 55.

### 3.2. Foam cell and lipid core

The key features of vulnerable plaques include a prominent lipid core and a high density of foam cells. The lipids within the plaque predominantly originate from the circulating blood. Under pathological conditions, the function of vascular ECs is impaired, leading to increased endothelial permeability. Circulating LDL infiltrates the vascular intima, which is oxidized and modified into ox-LDL.

Macrophages recruited and differentiated from the circulation can uptake ox-LDL via SR, subsequently transporting it to lysosomes where cholesterol esters undergo hydrolysis into free cholesterol and fatty acids 14, 56 (Figure 3B). For instance, M1 macrophages upregulate the expression of SR, including LOX-1, CD36, and SR-A, which exacerbate cholesterol absorption and ox-LDL 14, 56. Moreover, the impairment of the macrophage reverse cholesterol transport (RCT) cholesterol efflux pathway mediated by ABCA1 and ABCG1, leads to the accumulation of free cholesterol (FRC) in macrophages, ultimately contributing to foam cell formation 57. These FRCs spontaneously self-assemble and form cholesterol crystals (CCs). CCs can be released into the cytosol, inducing the complement cascade and activating the nucleotide-binding oligomerization domain-like receptor protein 3 (NLRP3) inflammasome, triggering inflammation via IL-1β production and causing macrophage apoptosis 58. Studies have shown that CCs are present at higher levels in the plaques of patients with acute coronary syndrome, indicating an association with plaque vulnerability and instability 59, 60. Therefore, many strategies currently focus on reducing the risk of plaque rupture by inhibiting foam cell formation 61, 62.

### 3.3. Pro-inflammation

Atherosclerosis is primarily characterized by a sustained imbalance between pro-inflammatory and anti-inflammatory factors. This imbalance contributes to the lesions deterioration, leading to macrophage death and impaired resolution of inflammation 63, 64. Macrophages play a crucial role in the progression of atherosclerosis. Inflammatory and immune responses are initiated when macrophages recognize pathogen-associated molecular patterns (PAMPs) and damage-associated molecular patterns (DAMPs) binding to their pattern recognition receptors. This interaction produces various pro-inflammatory molecules, including cytokines like interleukin-α, interleukin-β, and TNF (Figure 3D) 65.

Macrophage phenotypes at inflammatory sites are diverse, with differentiation into specific subtypes occurring in response to microenvironmental changes. In atheromatous plaques, macrophages are classified into M1 (pro-inflammatory phenotype) and M2 (anti-inflammatory phenotype) macrophages 66. Different subtypes of macrophages exhibit similar or opposing physiological functions and effects on the atherosclerotic environment. At rupture sites in vulnerable plaques, M1 phenotype macrophages contribute to plaque destabilization 67. Macrophages in plaques can be activated by IFN-γ, TNF-α, lipopolysaccharide, and TLR4 68. Notably, M1 macrophages express various pro-inflammatory factors, including TNF-α, IL-1β, IL-12, IL-23, and chemokines such as CXCL9, CXCL10, and CXCL11. These factors can activate Th1 and Th17 cells, producing elevated levels of nitric oxide (NO) and reactive oxygen species (ROS), contributing to severe vascular inflammation and exacerbating atherosclerosis. Additionally, macrophage derivatives, such as 25-hydroxycholesterol (25-HC), have been demonstrated to promote the development of vascular inflammation and atherosclerosis 69. Inhibiting the macrophage inflammatory response represents a crucial strategy for treating atherosclerosis. For example, a recent study showed that suppressing macrophage inflammasome activation with desmosterol prevented vascular inflammation and disease progression 70. In addition, some miRNAs, including miR-375 71, miR-30c-5p 72 and microRNA-99a/146b/378a 73 have been shown to inhibit macrophage inflammation and thus suppress the progression of atherosclerosis.

The inflammatory response mediated by macrophages is integral to the entire development stages of atherosclerosis. A deeper understanding of the pathological role of macrophages in this inflammatory response may offer valuable insights for potential atherosclerosis treatments.

### 3.4. Form necrotic core

An accumulation of lipids and cellular debris characterizes the necrotic core. The accumulation of necrotic cells and their contents contributes to forming and enlarging this core within plaques 74. In advanced human atherosclerotic plaques, 80% of necrotic cores exceed 1 mm², comprising over 10% of the total plaque area. Notably, in 65% of plaque ruptures, the necrotic core accounted for more than 25% of the area, indicating a significant correlation between core size and plaque vulnerability 75. Furthermore, the necrotic core in advanced plaques exhibits increased cholesterol clefts, abundant cellular debris, loss of ECM, and elevated tissue factor levels 76.

The prevalence of macrophage debris within the necrotic core has led to the hypothesis that plaque necrosis results directly from post-apoptotic macrophage death 77 (Figure 3E). Macrophages programmed cell death in atherosclerotic plaques encompasses apoptosis, necroptosis, pyroptosis, and ferroptosis. These forms of programmed cell death are interconnected components, with individual pathways closely linked and capable of compensating for one another 78. Macrophage death in advanced plaques triggers a series of physiological consequences, including the formation of apoptotic bodies and the release of cytokines and DAMPs, which contribute to plaque instability 79, 80. One prominent DAMP released in substantial quantities by late-stage macrophages is the HMGB-1 (high mobility group box 1) protein 81. Empirical studies indicate that HMGB-1 expression increases as atherosclerosis progresses. Neutralizing HMGB-1 inhibits atherosclerosis by reducing the accumulation of immune cells and migrating macrophages in the aortic plaques of ApoE^-/-^ mice 81, 82. Mechanistic studies demonstrate that HMGB-1 interacts with various receptors, including RAGE (receptor for advanced glycation end products), and activates NF-κB (nuclear factor-κB) signaling to induce inflammatory response. This process promotes the further development of plaques 83. Generally, macrophage death significantly influences the enlargement of the necrotic core. A comprehensive exploration of the mechanisms underlying programmed macrophage death could lead to the development of new therapeutic strategies for atherosclerosis.

### 3.5. Regulate neovascularization

A key distinction between vulnerable and stable plaques is the increased prevalence of neovascularization in the former, resulting in frequent plaque hemorrhages. The primary mechanism underlying this process is the induction of endothelial cell sprouting 84.

In vulnerable plaques, pathological neovascularization exhibits distinct morphological and functional characteristics, including abnormal vascular permeability, lack of a basement membrane and peripheral cells, and disorganized morphology 85, 86. Additionally, neovascularization in plaques often causes leakage and the release of intra-plaque erythrocytes, resulting in hemorrhage. Several studies suggest that neovascularization promotes macrophage infiltration in vulnerable plaques under pathological conditions 87 (Figure 3F). In other tissues, however, macrophages of various phenotypes have been shown to regulate angiogenesis 88, 89. However, clear evidence is lacking to demonstrate that macrophages directly regulate angiogenesis within plaques. Studies suggest a link between hypoxia-inducible transcription factors and macrophages in human atherosclerotic plaques and intra-plaque angiogenesis 90. Furthermore, Li *et al.* conducted non-invasive imaging of 32 subjects with carotid artery stenosis using FDG positron emission tomography and dynamic contrast-enhanced magnetic resonance imaging (MRI) before carotid endarterectomy. They discovered a strong association between the extent of neovascularization, plaque macrophage infiltration, and the expression of plaque major histocompatibility complex (MHC) II, a marker of plaque inflammation 91.

In conclusion, macrophages have a role in intraplaque neovascularization, and targeting macrophages may be necessary for preventing intraplaque hemorrhage and stabilizing plaques.

### 3.6. Regulate intraplaque hemorrhage

Most intraplaque hemorrhage (IPH) is associated with centripetal neovascularization originating from the epicardium towards the plaque and increased vascular wall permeability 92, 93. Recent studies have identified IPH as a contributing factor in forming vulnerable plaques 94, which are hallmark features of such plaques 95.

The correlation between IPH and macrophages plays a crucial role in the development of vulnerable plaques. For example, IPH can attract and release MMPs, destabilizing the plaque and accelerating rupture 96, 97. Beyond its effects on macrophage activity and plaque development, IPH is also subject to regulation. CD163, a marker of M2-type macrophages, has been demonstrated to promote angiogenesis and increase vascular permeability 98, while also being linked to atherosclerotic inflammation. IPH is strongly associated with thrombus formation, which exacerbates plaque instability 99, ultimately leading to conditions such as cerebral hemorrhage and stroke 100. Therefore, studying IPH could represent a breakthrough in treating vulnerable plaques. (Figure 3G).

### 3.7. Interact with other cell types within plaque

#### 3.7.1. VSMC phenotypic transformation

Most VSMCs exhibit a contractile phenotype in healthy vessels but can be converted to a synthetic phenotype in response to pathological injury. Historically, VSMCs within vulnerable plaques were considered entirely beneficial to the disease due to their ability to produce ECM, thus stabilizing the fibrous cap. However, recent discoveries have revealed that VSMCs can undergo a phenotypic transformation, influenced by various factors, leading to the emergence of osteoblast-like VSMCs, macrophage-like VSMCs, mesenchymal cell-like VSMCs, and other phenotypes, ultimately contributing to plaque instability 101.

In the atherosclerotic microenvironment, macrophages and VSMCs are in close proximity and interact with each other through direct contact or the release of soluble factors and active carriers, significantly influencing the progression of the disease 102 (Figure 3H). Dysfunctional macrophages within the plaque release pro-inflammatory mediators such as TNF-α, IL-1β, and IL-6, which lead to VSMC dedifferentiation, ECM degradation, and fibrous cap thinning 103. *In vitro* studies have demonstrated that co-culturing phorbol myristate acetate (PMA)-activated macrophages with human aortic VSMCs significantly reduces the synthesis of collagen type I and type III in VSMCs while simultaneously increasing the expression of matrix metalloproteinases (MMP1/9) 104. This diminishes plaque stability from two key perspectives: first, by reducing collagen synthesis, and second, by degrading the ECM components of the fibrous cap. Another study showed that inflammatory signaling (NLRP3) was activated in VSMCs after co-culturing VSMCs with ox-LDL-induced monocytes 105. NLRP3 has previously been shown to play a critical role in atherosclerosis and to facilitate the transformation of VSMCs into a macrophage-like phenotype. Additionally, extracellular vesicles (EVs) may represent a novel paradigm for interacting with macrophages and VSMCs. Although clear evidence of a regulatory effect of macrophage-derived EVs on VSMCs is lacking, sequencing and analysis of these vesicles indicated that their enriched contents may be associated with the migration and phenotypic transformation of VSMCs.

#### 3.7.2. Endothelial dysfunction

In addition to VSMCs, macrophages can also regulate the function of vascular endothelial cells in various ways (Figure 3H). For example, a study found that CD163 macrophages promoted vascular cell adhesion molecule (VCAM) expression and inflammation in plaques by inhibiting prolyl hydroxylase and activating hypoxia-inducible factor 1-alpha (HIF-1α) 98. Recently, a new study identified macrophage-promoted endothelial inflammation as a crucial factor in cholesterolemia-induced atherosclerosis 106. Their single-cell RNA-seq study of human atherosclerotic lesions revealed that sterol 27-hydroxylase (CYP27A1) was highly expressed in foam cells. CYP27A1 catalyzes the conversion of cholesterol into 27-hydroxycholesterol (27HC) and cholesteryl esters. Macrophage-derived CYP27A1-generated 27HC drives vascular inflammation and promotes atherosclerosis through estrogen receptor (ER) α ligands 106. In addition to the paracrine system, macrophages can regulate endothelial cell function by secreting EVs. For example, studies have shown that miRNAs enriched in macrophage-derived EVs can inhibit ECs proliferation^107^ and damage^108^.

#### 3.7.3. Activate T cell

Crosstalk between macrophages and T cells is essential for the progression of inflammatory atherosclerosis 109 (Figure 3I). Inflammation in atherosclerosis is initiated by releasing IL-1β, TNF, CCL2 and CCL5 from macrophages in response to innate immune activation. This inflammatory process is further promoted by the recruitment of monocytes, dendritic cells, and effector T cells (Th1 cells, Th17 cells, and Th2 cells). Effector T cells release inflammatory factors, amplifying the inflammatory response 110. Although macrophages can activate T cells as antigen-presenting cells, which is a fundamental aspect of acquired immune response activation 111, there is limited research on the mechanisms by which macrophages activate T cells in atherosclerosis. One study demonstrated that macrophage CD40 deficiency resulted in a bias towards the M2 phenotype, and CD40^-/-^ApoE^-/-^ mice had reduced CD4^+^ and CD8^+^ T cells, suggesting that macrophage CD40 deficiency can regulate T cell activation 112. Additionally, when miR-33-inhibited macrophages (which upregulated M2 phenotype markers) and naive T cells were co-cultured for 6 days, FoxP3^+^CD4^+^ Treg cells increased significantly, indicating a link between macrophages and innate adaptive immunity in atherosclerosis 113, 114.

## 4. Macrophage-targeted diagnosis for vulnerable plaques

### 4.1. Diagnostic imaging tools

Atherosclerotic plaque rupture and subsequent thrombosis are important causes of acute coronary syndrome (ACS), highlighting the essential need for the detection of vulnerable plaques in the management of atherosclerosis and CVD. A variety of both invasive and non-invasive imaging techniques have been developed for vulnerable plaque detection. Non-invasive methods include coronary angiography (CTA), MRI, and PET (positron emission tomography), while invasive methods include coronary angiography, intravascular ultrasound, optical coherence tomography, and near-infrared spectroscopy. The literature has extensively reviewed diagnostic imaging techniques for vulnerable plaques elsewhere 115. This section will specifically focus on imaging methods that target the unique characteristics of macrophages within vulnerable plaques.

Although less commonly utilized, MRI is an effective modality for characterizing crucial features of vulnerable carotid atherosclerotic plaques. The clinical application of commercial dextran-coated ultra-small superparamagnetic iron oxide nanoparticles (USPIOs), with a particle size of 30 to 50 nm, as T2 contrast agents has been reported for identifying atherosclerotic plaques in humans 116. Studies indicate that USPIOs are effectively phagocytosed by macrophages *in vivo*, resulting in detectable focal signal loss on MRI, which correlates with the extent of inflammation in atherosclerotic plaques. Consequently, USPIO-enhanced molecular MRI has been used to quantify the macrophage burden in plaques 117 and to evaluate plaques at risk of rupture in patients 118.

PET is a non-invasive imaging technique that uses contrast agents to evaluate plaque stability. PET radiotracers can identify activated macrophages and necrotic core microcalcifications, markers of vulnerable plaques. These contrast agents comprise ^18^F-sodium fluoride (^18^F-NaF, a marker of necrotic core microcalcifications), ^18^F-fluorodeoxyglucose (^18^F-FDG; a marker of activated macrophages), technetium-99m-annexin V (a marker of apoptosis cells), ^18^F-galacto RGD (a marker of angiogenesis), and ^68^Ga-DOTATATE (activated macrophages marker) 119. Among these, ^68^Ga-DOTATATE is notable for its specific recognition of inflammatory macrophages. Due to the specific expression of the SST2 gene by pro-inflammatory macrophages in vulnerable carotid plaques, ^68^Ga-DOTATATE serves as a tracer for vulnerable plaques, targeting inflammatory macrophages specifically 120. OCT is an invasive coronary artery imaging technique with an axial resolution of approximately 10-20 μm, excels in accurately measuring the true thickness of the fibrous cap. OCT can accurately differentiate between lipid and fibrous tissue and enables the visualization of macrophages, neovascularization, plaque rupture, and thrombosis 121. Preclinical studies have demonstrated that combined contrast agents can image macrophage burden in vulnerable plaques. These contrast agents include iodinated and gold nanoparticles 122, 123.

Non-invasive optical imaging techniques, such as optical fluorescence and bioluminescence imaging, can be used to identify vulnerable atherosclerotic plaques. Since most of this research is still preclinical biomedical imaging research, we will discuss this method in the following paragraphs.

### 4.2. Novel targets

**Figure 4:**
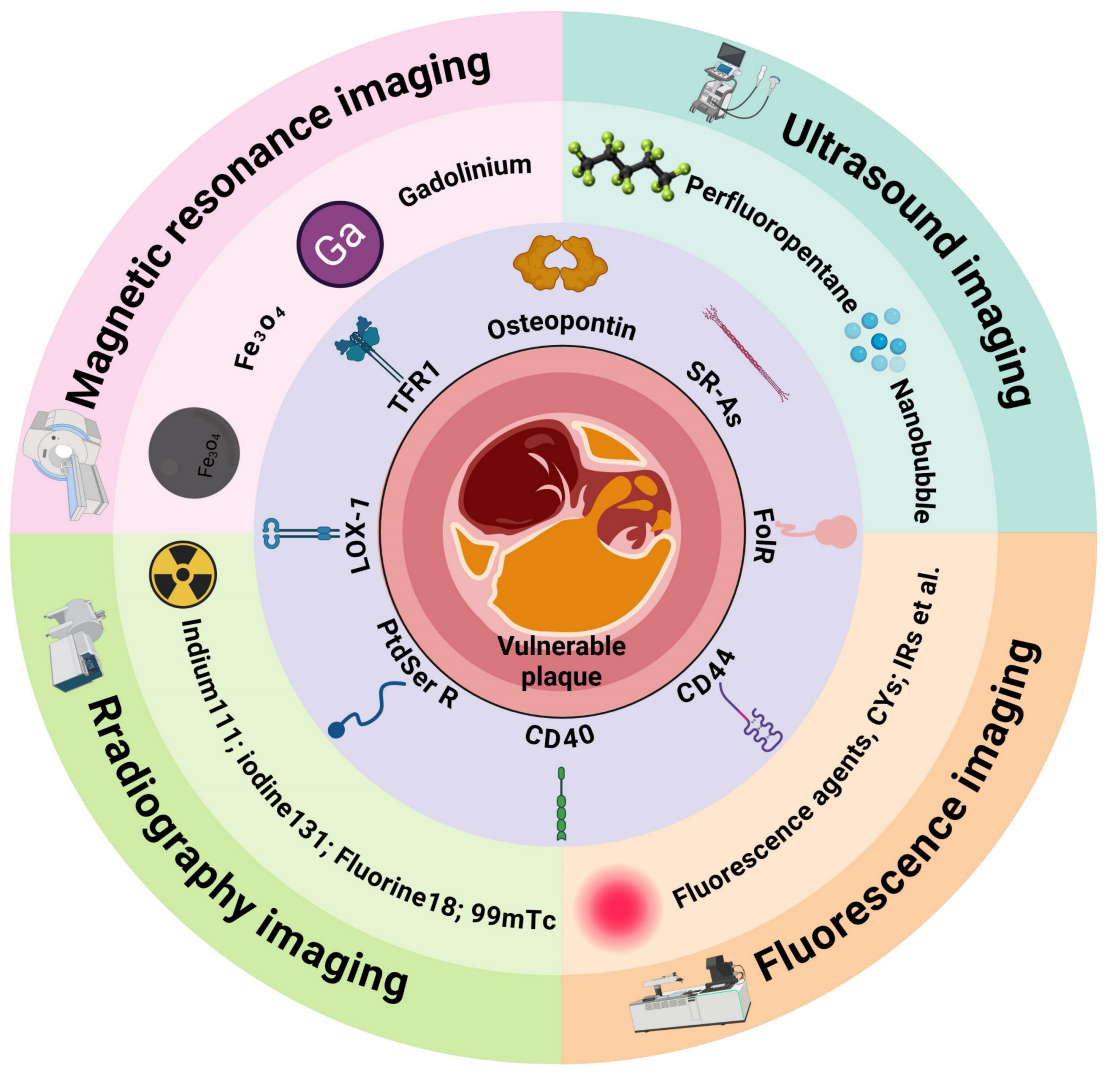
** A schematic overview of the novel targets and the imaging agents used to diagnose vulnerable atherosclerotic plaques.** TFR1 = t*ransferrin receptor 1*; SR-As = *scavenger receptors A*; FolR = f*olate receptor*; PSR =* phosphatidylserine receptor*. *Figure created with BioRender.com*.

Given the central role of macrophages in plaque deterioration, their associated pathological processes are crucial targets for diagnostic imaging of vulnerable plaques 124. In recent years, nanotechnology-based imaging techniques targeting macrophages have enabled an accurate plaque vulnerability assessment (Figure 4). This section will primarily summarize the imaging strategies of nanoplatforms targeting macrophages in vulnerable plaques (Table 1).

#### 4.2.1. Osteopontin

Osteopontin (OPN) is a secreted protein that functions as a chemotactic cytokine, facilitating the adhesion, migration, and activation of foam macrophages. Studies have demonstrated foamy macrophages in human atherosclerotic lesions overexpression OPN, a finding confirmed through histological and molecular analyses 125. Consequently, several strategies have been developed to evaluate plaque vulnerability by targeting OPN in foam macrophages. For instance, Zhen *et al.* conjugated OPN antibodies to Ti_3_C_2_ nanoplates and loaded them with indocyanine green (ICG) to enable photoacoustic imaging of vulnerable plaques 126. The OPN Ab/Ti_3_C_2_/ICG nanoprobes demonstrated exceptional targeting capabilities for foam cells and vulnerable atherosclerotic plaques in both *in vitro* and *in vivo* studies 126. More recently, Zhang *et al.* developed an atherosclerosis-targeted nanoprobe utilizing near-infrared (NIR) persistent luminescent nanoparticles (PLNPs) to detect early vulnerable plaques 127. The surface conjugation of anti-OPN antibodies significantly enhanced the nanoprobe's targeting ability for foam cells. Upon intravenous administration in atherosclerosis model mice, this sensitive nanoprobe successfully detected atherosclerotic plaques prior to ultrasound (US) and MRI, achieving a signal-to-noise ratio (SNR) as high as 5.72 127.

#### 4.2.2. Scavenger receptors A (SR-As)

SR-A is a transmembrane glycoprotein receptor abundantly expressed on activated macrophages within vulnerable plaques. Consequently, several nano-delivery strategies have been developed to visualize vulnerable plaques by targeting SR-A. For example, Chen *et al.* conjugated SR-A-targeted dextran sulfate to PLGA-PEG-PLGA nanoparticles and loaded them with fluorescein DiR (1,1′-dioctadecyl-3,3,3′,3′-tetramethylindotricarbocyanine iodide) and Fe_3_O_4_. This nanocarrier targets macrophages in vulnerable aortic arch plaques, enabling near-infrared fluorescence (NIRF) and MRI of these plaques 128. Another commonly utilized SR-A-targeted ligand is the PP1 peptide (LSLERFLRCWSDAPAK), known for its high affinity and specificity for SR-A. Zhang *et al.* synthesized mesoporous silica nanoparticles loaded with NIR dye IR820 and Fe_3_O_4_, subsequently conjugating them with the PP1 peptide. Fe_3_O_4_ was a magnetic core for T2 and T2*-weighted MR imaging, while IR820 was used for optical imaging. This carrier, featuring two composite imaging elements, enables dual-modality imaging of vulnerable atherosclerotic plaques 129.

#### 4.2.3. Transferrin receptor 1

Transferrin receptor 1 (TFR1, CD71) is a type II transmembrane glycoprotein that binds transferrin and plays a crucial role in cellular iron uptake by interacting with iron-bound transferrin. Studies have demonstrated that the expression of the transferrin receptor is markedly elevated in vulnerable plaques, coinciding with the accumulation of heavy-chain ferritin (HFn) in infiltrating macrophages within human atherosclerotic plaques 130, 131. To address this, Yan *et al.* developed radiolabeled bioengineered ^99m^Tc-H-Ferritin nanocages to target TFR1 and visualize vulnerable plaques. Their study revealed that the iron-storing nanocage HFn is selectively absorbed by infiltrating macrophages in high-risk and early atherosclerotic plaques, enabling highly sensitive imaging of vulnerable and early active plaques 132. The OX26 antibody has also been utilized as a ligand for targeting TFR1 in tumor delivery and imaging 133, 134. However, this strategy has not yet been implemented in vulnerable atherosclerotic plaques.

#### 4.2.4. Phosphatidylserine receptor

Under normal conditions, most phosphatidylserine (PtdSer) is localized to the inner leaflet of the cell membrane. However, as atherosclerosis advances, extensive apoptosis leads to the translocation of PtdSer to the outer layer of the cell membrane 135. Macrophages express PtdSer receptors on their surface, enabling them to recognize and eliminate apoptotic cells 136. This makes PtdSer receptors an ideal target for imaging vulnerable plaques. Mikako *et al.* loaded PEG-modified PtdSer liposomes with radioactive ^111^In, enabling PET imaging of macrophages in atherosclerotic plaques 137. In addition, they designed a cathepsin B (CTSB)-responsive peptide-IGG fluorescent probe, which was encapsulated into PtdSer liposomes 138. This carrier can target macrophages infiltrating plaques and emits fluorescence upon CTSB activation, facilitating NIRF imaging 138.

#### 4.2.5. Folate receptor

The folate receptor beta (FolR-β) expression in activated macrophages 139 indicates its potential as a specific marker for vulnerable plaques. Researchers have employed folate-based radiopharmaceuticals (such as ^111^In-EC0800) combined with high-resolution animal single-photon emission computed tomography (SPECT/CT) for distinguishing between stable and vulnerable plaques 140. Additionally, nano-molecules have been developed by conjugating the fluorescent contrast agent fluorescein isothiocyanate (FITC) with folate ligands to evaluate plaque vulnerability 141. Furthermore, different research employed aluminum fluoride-18-labeled folic acid to visualize *in vivo* inflammation of atherosclerotic plaques through positron emission tomography 142. In a recent study, Guo *et al.* developed a range of radio-iodinated albumin compounds targeted at folate receptors to image vulnerable plaques 143. Among these compounds, [^131^I]-IBNHF stands out as a promising radiotracer for SPECT imaging of atherosclerosis 143.

Additionally, several macrophage-specific receptors, such as lectin-like oxidized low-density lipoprotein receptor 1 (LOX-1), CD44, and CD40, have been utilized as targets for imaging vulnerable plaques.

#### 4.2.6. Phagocytosis

In addition to the previously mentioned macrophage-specific targets, macrophage phagocytosis can also be utilized to image vulnerable plaques. The semiconductor polymer-based nanoparticles (RSPN) developed by Zhang *et al.* enable highly specific and reliable imaging of O2•- in vulnerable plaques 144. RSPN nanoparticles demonstrate excellent photoacoustic imaging capabilities and can be phagocytosed by macrophages *in vivo* and *in vitro*, allowing for accurate assessment of pneumonia-induced atherosclerotic vulnerable plaques 144.

## 5. Macrophage-targeted therapeutic strategy for stabilizing vulnerable plaques

**Figure 5:**
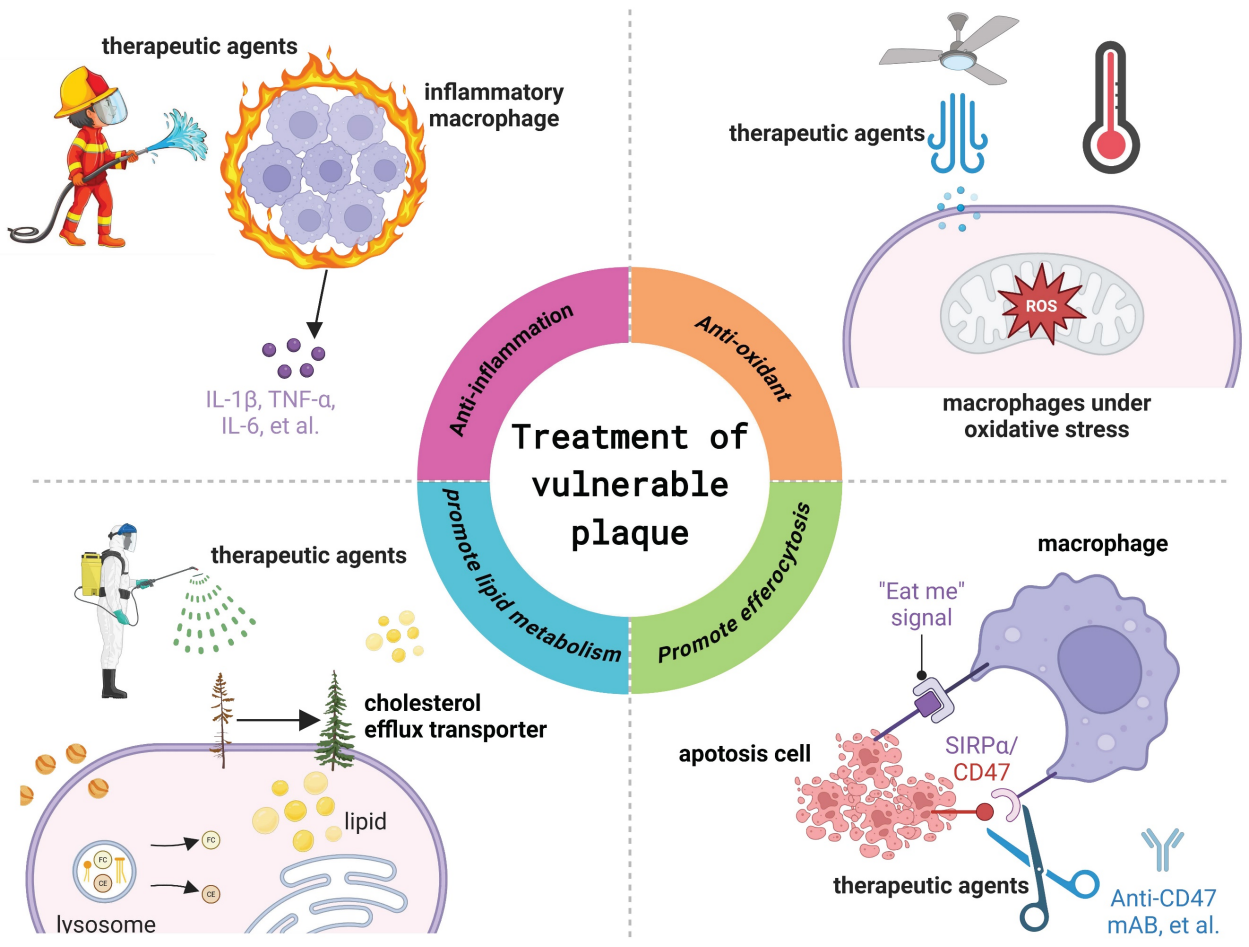
** Macrophage targeted therapeutic strategy for the treatment of vulnerable atherosclerotic plaques.** *Figure created with BioRender.com*.

Given the pivotal role of macrophages in the progression of vulnerable plaques, research efforts increasingly focus on modulating macrophage function as a therapeutic strategy for treating atherosclerosis and stabilizing vulnerable plaques. Present strategies aiming to regulate macrophage function to improve the stability of vulnerable plaques mainly involve inhibiting macrophage inflammation, modulation of lipid metabolism in macrophages and foam cells, and promotion of macrophage exocytosis (Figure 5).

### 5.1. Anti-inflammation

There is a widespread consensus that atherosclerosis represents a chronic inflammatory malady that impacts blood vessels 52. Consequently, anti-inflammatory therapy is an indispensable approach for managing atherosclerosis. For instance, the CANTOS study led by Dr. Paul Ridker demonstrated that subcutaneous injection of 300 mg of canakinumab, an IL-1β monoclonal antibody, every three months could reduce high-sensitivity C-reactive protein levels by 41% from baseline and lower the risk of cardiovascular events, the primary endpoint, by 14% 165. This study provides direct evidence supporting the inflammatory hypothesis of atherosclerosis and serves as a foundation for the development of targeted anti-inflammatory therapies. Another notable case is colchicine, which has been approved by the U.S. Food and Drug Administration for the anti-inflammatory treatment of cardiovascular disease. A multinational, randomized, double-blind, placebo-controlled phase 3 clinical trial involving 5,522 patients with chronic coronary heart disease revealed that colchicine, when added to high-dose statins and other standard preventive therapies (0.5 mg daily), reduced the overall risk of cardiovascular death, spontaneous myocardial infarction, ischemic stroke, or ischemia-driven coronary artery revascularization by 31% compared to the placebo group 166. Additionally, a recent prospective, single-center, randomized, double-blind clinical trial involving 128 patients with acute coronary syndrome found that 12 months of colchicine therapy (0.5 mg daily) significantly improved optical coherence tomography (OCT) parameters, including fibrous cap thickness (FCT), lipid arcs, and macrophage infiltration 167. Macrophages are the primary effector cells in the inflammatory process within plaques and play a crucial role in innate and adaptive immunity 168. Therefore, various medications are delivered to plaque macrophages to exert anti-inflammatory effects and stabilize plaques, including rapamycin 169, ibrutinib 170, and quercetin 171, among others.

Additionally, the repolarization of macrophage phenotypes has emerged as a novel approach for the anti-inflammatory treatment of atherosclerosis. Given the phenotypic flexibility of macrophages within plaques, reprogramming inflammatory M1 macrophages into anti-inflammatory M2 phenotypes can assist in inhibiting atherosclerosis and enhancing plaque stability 172, 173. For example, Zhang *et al.* devised ROS-responsive dextran-g-PBMEO NPs with biomimetic modification of macrophage membranes to reprogram macrophage phenotypes by delivering the anti-inflammatory drug kaempferol specifically to plaques 174. Their study confirmed that KPF@MM-NPs treatment significantly reduced inflammatory-related proteins, decreasing p-IκB levels by 3.2-fold and p-p65 levels by 1.2-fold in RAW264.7 macrophages compared to the model group. *In vivo* experiments further demonstrated that KPF@MM-NPs reduced the aortic plaque area to approximately 2.81% of the total aortic tissue area. Additionally, the treatment increased the collagen content and the number of VMCs surrounding the plaque, indicating improved plaque stability 174. Recently, EVs have shown great potential in disease treatment due to their rich content and natural immunogenicity 175. Our group stimulated endothelial cells to produce EVs with high anti-inflammatory activity (LSS-EVs) through hydrodynamics, which can significantly inhibit the progression of atherosclerosis by reprogramming macrophages to the anti-inflammatory M2 phenotype *in vivo* 173.

### 5.2. Anti-oxidant

Numerous studies have demonstrated that oxidative stress promotes atherosclerosis 176. The accumulation of ROS represents a primary pathological consequence of oxidative stress. ROS can induce macrophage activation, cell apoptosis, and oxidative damage to biological macromolecules (e.g., lipids, proteins, DNA) 149. ROS and its oxidative products can activate various signaling pathways involving functional signaling molecules, leading to changes in vascular cells and impacting vascular function and structure. Therefore, clearing ROS within atherosclerotic plaques is crucial for treating atherosclerosis. Gu *et al.* identified that Prussian blue nanozyme (PBNZ) demonstrates outstanding enzymatic activity and effectively eliminates ROS 177. Consequently, they meticulously designed a PBNZ-based nanoparticle (PBNZ@PP-Man) to stabilize vulnerable plaques 177. Their study revealed that PBNZ@PP-Man was internalized by macrophages via mannose receptor-mediated endocytosis, effectively alleviating inflammation by clearing ROS and enhancing endocytosis 177. In *in vivo* animal experiments, the plaque area at the arterial root of mice treated with PBNZ@PP-Man decreased by 56.6% and collagen production was 2.6 times higher than that of the untreated control group 177.

He *et al.* recently developed two-dimensional black phosphorus nanosheets functionalized with Resolvin D1 (BPNSs@PEG-S2P/R) to effectively eliminate ROS within plaques to treat atherosclerosis 178. Their research revealed that BPNSs@PEG-S2P/R efficiently accumulated within the diseased macrophages of atherosclerotic plaques aided by the S2P peptide. Furthermore, Resolvin D1 was released under ROS stimulation to eliminate ROS and suppress the ROS-induced inflammatory response in macrophages. Moreover, BPNSs@PEG-S2P/R can eliminate ROS within plaques *in vivo*, while increasing collagen content in the aortic peduncle reduces the incidence of ruptured or buried fibrous caps in the brachiocephalic artery region of mice. Additionally, it increases fibrous cap thickness.

### 5.3. Regulate lipid metabolism

Under normal conditions, macrophages can process substantial amounts of lipids and cholesterol in the blood vessel wall without significantly overburdening the metabolic decomposition process 179. In the context of atherosclerosis, the capacity of macrophage lipid metabolism diminishes, leading to disrupted cholesterol and lipid metabolism, as well as cell apoptosis and necrosis. These necrotic macrophages are a significant factor influencing plaque stability. Therefore, restoring macrophage lipid transport capacity can help prevent the progression of atherosclerosis and maintain plaque stability. ABCA1, ABCG1, and SR-B1 play critical roles in cholesterol reverse transport. Liver X receptor (LXR) agonists (such as GW3965 and T0901317) are among the commonly used drugs for regulating macrophage lipid metabolism in atherosclerosis. For example, the LXR agonist GW3965 can upregulate the reverse cholesterol transporter ABCA1 expression in the small intestine and peripheral macrophages, elevate circulating HDL levels, and prevent atherosclerotic cardiovascular disease 180. Yu *et al.* utilized DSPE-PEG nanoparticles to encapsulate the LXR agonist GW3965, aiming to mitigate plaque formation by upregulating ABCA1, predominantly expressed in macrophages, to enhance intracellular phospholipid and cholesterol efflux while not influencing hepatic lipid metabolism 181. Recently, Chen *et al.* identified the significant role of Epsins in regulating lipid metabolism and transport in macrophages 182. They observed that Epsins bind to CD36 and enhance lipid uptake by promoting CD36 endocytosis and recycling 182. Conversely, Epsins facilitate ABCG1 degradation via lysosomes, impeding ABCG1-mediated cholesterol efflux and reverse cholesterol transport. Building on this model, they developed siRNA-loaded nano-therapies to inhibit Epsins, thereby halting inflammation and expediting the regression of atherosclerosis.

### 5.4. Promote efferocytosis

The accumulation of apoptotic cells in the necrotic core is a prominent feature of atherosclerotic plaques. Typically, apoptotic cells are eliminated through a process known as endocytosis, which is triggered by "eat me" ligands, signifying "entering the grave" 183. Conversely, cells may overexpress "do not eat me" ligands to evade elimination. Research has demonstrated that the CD47 protein, a member of the "do not eat me" family of molecules, impedes the phagocytosis of apoptotic cells through its interaction with the signal regulatory protein α (SIRPα) receptor on phagocytes 184. Thus, blocking the CD47 signal is a crucial strategy for controlling the elimination of apoptotic cells in atherosclerotic plaques. For example, Kojima *et al.* administered anti-CD47 antibodies and demonstrated an improvement in exocytosis within plaques and a reduction in the formation of necrotic cores 185. To improve the bioavailability of CD47 antibody therapy and mitigate side effects, Chen *et al.* encapsulated CD47 antibodies in mesoporous silica nanoparticles and modified them with platelet membranes to prolong blood circulation time 186. They discovered that this biomimetic carrier displayed immune evasion properties in both *in vitro* and *in vivo* settings, significantly reducing the plaque area of the aortic foot from 46.7 ± 2% to 16.6 ± 1.7% and increasing the VSMC content of the aortic foot from 22.1 ± 2.3% to 55.2 ± 4.1% 186. Furthermore, Flores *et al.* employed nanotubes loaded with a small molecule inhibitor targeting the anti-phagocyte CD47-SIRPα signaling axis (SWNT-SHP1i) to induce phagocytosis at the plaque site 187. Their findings validated the accumulation of SWNT-SHP1i in atherosclerotic plaques, reactivation of lesion phagocytosis, reduction of necrotic core and accumulation of apoptotic bodies in atherosclerotic mice, and enhancement of plaque stability 187. Recently, Jarr *et al.* discovered that statins can enhance programmed cell elimination by restraining the NF-κB1 and inhibiting the expression of the pivotal "do not eat me" molecule, CD47 188. Statins enhance the anti-atherogenic effects of macrophage phagocytosis in an additive fashion, irrespective of any lipid-lowering effects 188.

## 6. Conclusions and future perspectives

Atherosclerosis is a widespread global disease with high prevalence across all regions. It also serves as an underlying factor for other cardiovascular diseases, including stroke and heart attack. Vulnerable plaques represent high-risk manifestations of atherosclerosis and studying them can lead to breakthroughs in diagnosing and treating atherosclerosis. We reviewed the primary pathological characteristics of vulnerable plaques and summarized the methodologies for constructing *in vivo* mouse models of vulnerable plaques. Modifying the plaque's local hemodynamic environment is a widely employed strategy for constructing vulnerable plaques. Among these methods, the right carotid artery tandem ligation model is a promising standard model for inducing vulnerable plaques in mice, as it can form stable and vulnerable plaques in the adjacent tissues of the same individual. This is significant for preclinical studies on vulnerable plaque pathology and therapeutic diagnostics. In addition, there are reports that transgenic ApoE^-/-^ mice can develop vulnerable plaques. However, there may be potential drawbacks regarding model cost and stability. Despite exploring several relatively mature vulnerable plaque construction models in mouse models, there is still a lack of atherosclerosis and vulnerable plaque models in large animals.

Macrophages and macrophage-derived foam cells play an essential role in forming and rupturing vulnerable plaques. For example, macrophages can regulate ECM remodeling, lipid accumulation, inflammatory response, necrotic core formation, neovascularization, intraplaque hemorrhage, and the phenotypic transformation of smooth muscle cells. However, the formation of stable plaques is also accompanied by pathological processes such as lipid accumulation, inflammation, and the formation of necrotic cores. Therefore, it is still difficult to clearly define the difference between the functions of macrophages in stable plaques and those in vulnerable plaques. Since plaque rupture is a multifactorial pathological process, exploring the interaction and synergy between macrophages and other factors influencing vulnerable plaques is necessary.

Considering the important role of macrophages in vulnerable plaques, macrophage-based therapeutic diagnostics represent a logical and highly promising strategy for identifying and preventing the development of vulnerable plaques. Most macrophage-based diagnostic strategies for vulnerable plaques are in the preclinical research stage. These studies primarily focus on screening targets of macrophages in vulnerable plaques and innovating contrast agents. Currently, the reported macrophage targets that can identify vulnerable plaques include OPN, SR-As, transferrin receptor 1, phosphatidylserine, folate receptor, and others. Nanocarriers that bind to these targets and load-effective imaging agents can be used to assess the risk of vulnerable plaques. However, it should be noted that most of these studies compare imaging differences between vulnerable plaques and healthy tissues while ignoring the comparison with stable plaques. Therefore, developing strategies for achieving high-resolution imaging of vulnerable plaques that differ from stable plaques is essential.

Similarly to the imaging strategy for vulnerable plaques, the nano-drug delivery strategy is also in the preclinical research stage. Presently, strategies aimed at targeting macrophages to prevent and stabilize vulnerable plaques focus on inhibiting macrophage inflammation antioxidation, regulating macrophage cholesterol metabolism, and promoting macrophage endocytosis. Nevertheless, these pharmacological concepts for treating vulnerable plaques are nearly identical to the conventional pharmacological concepts for inhibiting the progression of atherosclerosis. Consequently, an in-depth exploration of the pathological mechanisms underlying vulnerable plaque formation, development, and rupture will significantly advance drug development targeting vulnerable plaques.

To conclude, this review outlines the pathologic features and animal models of vulnerable atherosclerosis plaques and summarizes the pivotal role of macrophages in their development. Furthermore, we also discuss the targeted diagnosis and treatment strategies for vulnerable plaques, focusing on the role of macrophages. Gaining a deeper understanding of the pathological mechanisms underlying vulnerable plaques and developing appropriate experimental animal models will significantly advance the field of effective treatment and diagnosis of atherosclerosis and simultaneously address related clinical issues.

## Figures and Tables

**Table 1:** Macrophage-based vulnerable plaque imaging system

Targets	Ligand	Carrier	Imaging agents	Imaging type	Year
Osteopontin	Osteopontin antibody	Fe_3_O_4_ NP	Fe_3_O_4_/Cy-5.5	Fluorescence/MRI	2017 125
	Osteopontin antibody	NaGdF_4_:Yb,Er@NaGdF_4_	NaGdF_4_	MRI	2017 145
	Osteopontin antibody	Ti_3_C_2_ nanosheets	Indocyanine green	PA	2020 126
	Osteopontin antibody	Mesoporous silicon NP	Au NP	US/Fluorescence/ Photothermal	2023 146
	Osteopontin antibody	mSiO_2_ NP	NIR persistent luminescent material	Fluorescence	2024 127
	antiOPN peptide	Human serum albumin NP	IR780	Fluorescence/MRI	2022 147
SR-As	Dextran sulfate	PLGA NP	Fe_3_O_4_/perfluoropentane	US/MRI	2019 148
	Dextran sulfate	PLGA-PEG-PLGA NP	Fe_3_O_4_/DiR/perfluoropentane	Fluorescence/MRI	2022 128
	Dextran sulfate	Prussian blue-PEI	Rhodamine/Gd	Fluorescence/MRI	2022 149
	PP1 peptide	Mesoporous silica NP	IR820/iron oxide NP	Fluorescence/MRI	2021 129
	PP1 peptide	PLGA NP	Fe_3_O_4_/perfluoropentane	US/MRI	2021 150
Transferrin receptor 1	High- Ferritin	Ferritin nanocages	^99m^Tc	Radiography	2018 132
PtdSer receptor	Phosphatidylserine	Phosphatidylserine liposome	Indocyanine green	Fluorescence	2019 138
	Annexin V	Nanobubble	-	US	2022 151
**Target**	**Ligand**	**Carrier**	**Imaging agents**	**Imaging type**	**Year**
	Peptide (CLIKKPF)	Cyclodextrin	Lipid target probes	Fluorescence	2024 152
Folate receptor	Folate	-	FITC	Fluorescence	2012 153
	Folate	-	^111^indium-EC0800	Radiography	2014 154
	Folate	-	^18^F	Radiography	2018 155
	Folate	Human serum albumin	^131^I	Radiography	2023 143
	Folate	Pd@Au	Pd/Au	CT/PA	2020 156
LOX-1	LOX-1 antibody	-	^99m^Tc	Radiography	2008 157
	LOX-1 antibody	-	^111^indium/Gd/DiL	SPECT/CT/MRI/ Fluorescence	2010 158
	LOX-1 antibody	Fe_3_O_4_ NP	Fe_3_O_4_	MRI	2014 159
CD44	Hyaluronic acid	Hybrid liposomal cerasomes	Gadodiamide	MRI	2022 160
	oxidized dextran	-	Lipid probe/iodinated contrast	Fluorescence/CT	2024 161
CD40		Fe_3_O_4_ NP	Cy5.5/iron oxide	Fluorescence/MRI	2023 162
Phagocytosis	-	Bovine serum albumin	Cy-3-NO_2_/Mito-NIRHP	PA	2018 163
	-	DSPE-PEG NP	Semiconducting polymer	PA	2021 144
	-	Amino acid NP	NIR dye	Fluorescence	2024 164

Abbreviations (NP: nanoparticles; MRI: magnetic resonance imaging; PA: photoacoustic; US: ultrasonic; NIR: near-infrared fluorophores; FITC: Fluorescein Isothiocyanate)
